# Stand-Alone Personalized Normative Feedback for College Student Drinkers: A Meta-Analytic Review, 2004 to 2014

**DOI:** 10.1371/journal.pone.0139518

**Published:** 2015-10-08

**Authors:** Keri B. Dotson, Michael E. Dunn, Clint A. Bowers

**Affiliations:** Department of Psychology, University of Central Florida, Orlando, Florida, United States of America; Leibniz Institute for Prevention Research and Epidemiology (BIPS), GERMANY

## Abstract

**Background:**

Norms clarification has been identified as an effective component of college student drinking interventions, prompting research on norms clarification as a single-component intervention known as Personalized Normative Feedback (PNF). Previous reviews have examined PNF in combination with other components but not as a stand-alone intervention.

**Objectives:**

To investigate the degree to which computer-delivered stand-alone personalized normative feedback interventions reduce alcohol consumption and alcohol-related harms among college students and to compare gender-neutral and gender-specific PNF.

**Data Sources:**

Electronic databases were searched systematically through November 2014. Reference lists were reviewed manually and forward and backward searches were conducted.

**Selection Criteria:**

Outcome studies that compared computer-delivered, stand-alone PNF intervention with an assessment only, attention-matched, or active treatment control and reported alcohol use and harms among college students.

**Methods:**

Between-group effect sizes were calculated as the standardized mean difference in change scores between treatment and control groups divided by pooled standard deviation. Within-group effect sizes were calculated as the raw mean difference between baseline and follow-up divided by pooled within-groups standard deviation.

**Results:**

Eight studies (13 interventions) with a total of 2,050 participants were included. Compared to control participants, students who received gender-neutral (*d*_between_ = 0.291, 95% CI [0.159, 0.423]) and gender-specific PNF (*d*_between_ = 0.284, 95% CI [0.117, 0.451]) reported greater reductions in drinking from baseline to follow-up. Students who received gender-neutral PNF reported 3.027 (95% CI [2.171, 3.882]) fewer drinks per week at first follow-up and gender-specific PNF reported 3.089 (95% CI [0.992, 5.186]) fewer drinks. Intervention effects were small for harms (*d*_between_ = 0.157, 95% CI [0.037, 0.278]).

**Conclusions:**

Computer-delivered PNF is an effective stand-alone approach for reducing college student drinking and has a small impact on alcohol-related harms. Effects are small but clinically relevant when considered from a public health perspective. Additional research is needed to examine computer-delivered, stand-alone PNF as a population-level prevention program.

## Introduction

Alcohol is one of the leading causes of death among individuals 15 to 24 years of age worldwide [1]. College students in this age range are a particularly high-risk group in need of effective prevention and intervention programs. National survey findings indicate that 80% of U.S. college students consumed alcohol in the past thirty days [2]. Compared to non-collegiate peers of the same age, college students engage in more high-risk drinking behaviors and experience more alcohol-related negative consequences, including alcohol-related sexual assault, injury, and death [2]. Alcohol use is also the most prevalent contributor to academic failure among college students, with nearly 25% of college students reporting alcohol-related academic consequences, including missed classes, incomplete assignments, and poor grades [2–4].

In addition to fatalities, physical harms, and academic consequences experienced by college students, the financial impact of drinking is enormous. According to the Center for Disease Control (CDC), the economic cost of alcohol misuse among adults in the United States in 2006 was $223.5 billion and included losses in workplace productivity, healthcare, criminal justice expenses, and motor vehicle accidents [5]. Nearly 76% of these alcohol-related costs were attributed to binge drinking, defined as consuming five or more drinks in one sitting for men and four or more drinks in one sitting for women [6]. In the U.S., binge drinking is more common among college students than non-collegiate peers, with 40% of U.S. college students reporting at least one binge drinking episode in the past two weeks [6–9]. In a national survey of U.S. college students, 13% reported engaging in “extreme binge drinking,” defined as consuming ten or more drinks in one sitting during the past two weeks [7]. Shockingly, 15% of emerging adults age 21 to 24 reported consuming 20 or more drinks in one sitting [7]. College students were also more likely to endorse a pattern of heavy episodic drinking–binge drinking periodically–rather than frequent, heavy drinking [7]. Each binge drinking episode constitutes high-risk drinking. At this high level of consumption, each additional drink is associated with increased risk for harms. Given this risk, even a small reduction in quantity consumed could reduce the risk of another alcohol-related fatality. This is a key premise for prevention and early intervention programs for college student drinking. As risky drinking and widespread negative consequences among college students continues to be a major public health concern, there is increasing emphasis on the growing need for universal prevention and intervention programs aimed at reducing high-risk drinking at both the population-level and the individual-level [10].

Social Norms Theory (SNT) provides the theoretical basis for social norms interventions. This theory posits that an individual’s perception of how peers think and act influences the individual’s behavior. There is substantial evidence implicating social norms as a contributory factor for high-risk drinking among college students. Research has shown that individual beliefs about peer alcohol use significantly predict personal alcohol use among college students [11–13]. Further, college students tend to overestimate peer alcohol use and subsequently increase their own alcohol use based on this overestimation [14, 15]. According to SNT, normative influence will lead to behavior change *only* when “highlighted prominently in consciousness” [16]. Drawing on this theory, social norms interventions aim to increase students’ awareness of their own drinking patterns and highlight any discrepancies between their own drinking patterns, their perceptions of peer drinking patterns, and actual peer drinking patterns. According to SNT, highlighting discrepancies in perceived and actual peer drinking and correcting normative misperceptions should lead to drinking reductions.

Social norms clarification has been examined as both a universal prevention program and as an individual intervention [17]. Most universal social norms approaches have been implemented through large-scale social marketing campaigns and have been found to have little or no effect on drinking norms, alcohol use, or alcohol-related harms (for a review see Foxcroft et al. [18]). It has been hypothesized that the impersonal nature of such campaigns may account for their poor effects. When provided in an individual format, interventions that include social norms clarification have shown moderate to large effects on drinking norms and small to moderate effects in reducing alcohol consumption and binge drinking episodes among high-risk college students [19–23]. Social norms clarification has typically been studied in combination with additional intervention components, but recent research has focused on social norms clarification as a stand-alone intervention without additional components, known as Personalized Normative Feedback (PNF) [22, 24–30].

PNF interventions typically use graphs and text to provide individualized feedback based on self-report measures of alcohol use (e.g., number of drinking days per week, average drinks per sitting, average drinks per week, etc.). Generally, feedback content is consistent across PNF interventions with any differences primarily being with format or aesthetic aspects [22, 24–30]. Feedback usually includes bar graphs to display a student’s own drinking behavior, their perceptions of drinking norms for a specified reference group, and actual drinking behavior for the specified reference group. These data for actual reference group drinking rates are typically derived from surveys administered at individual universities where PNF is being implemented. Students are also provided a percentile rank comparing their drinking behaviors with that of other students in their reference group (e.g., “Your percentile rank is 72%; this means that you drink as much or more than 72% of other college students on your campus”) [22, 24–30]. PNF reference groups may be general, known as gender-neutral (e.g., “college students on your campus”) or may be matched on gender or another demographic characteristic (e.g., gender-specific which might be “female college students on your campus”) [22, 24–30].

Most PNF interventions have used “typical students” as the normative reference group; however, recent research has suggested that referents of greater specificity (e.g., gender-specific) may increase PNF efficacy for particular groups [15, 26, 31]. In a study examining gender-specificity of normative referent, researchers found gender-specific PNF to be more effective than gender-neutral PNF for women, particularly among those who were higher in gender identity [15, 32]. This research has indicated that gender-specific and gender-neutral feedback may be differentially effective for women and men. Two reasons have been proposed for gender differences. First, there are actual gender differences in alcohol consumption such that men drink more than women, therefore female gender-specific norms are lower than gender-neutral norms and male gender-specific norms are higher. It has been hypothesized that gender-neutral norms will be more effective for men because these norms provide a greater discrepancy than gender-specific male norms. Second, research has found that women perceive the “typical college student” as including males and females, therefore, gender-specific feedback provides a more proximal referent [15, 32].

The efficacy of PNF has been evaluated in provider-guided and a variety of self-guided formats (e.g., mail, email, web-based) with promising results. PNF has been implemented in structured settings (e.g., research laboratory or clinical setting) as well as unstructured settings (e.g., self-guided web-based completion). PNF was introduced originally as a single component included in more extensive face-to-face provider-guided interventions and, more recently, as a component in computer-delivered interventions [23, 33]. As research on computer-delivered interventions has continued to expand, PNF has been examined as a stand-alone computer-delivered intervention. First, computer-delivered PNF was administered as a self-guided intervention in a structured setting, with results suggesting small to moderate effects for drinking reductions [24, 34]. More recently, computer-delivered stand-alone PNF has been evaluated as a web-based intervention in non-structured settings. Students typically receive an emailed link to access the intervention online and then complete the program when convenient [25, 26]. Whether administered in a structured or non-structured setting, computer-delivered PNF is the predominant modality at this time. In a review of individual interventions for college student drinking, small to moderate within-group effects on drinking were found for stand-alone PNF [35], and effects were more pronounced among college students who reported drinking for social reasons [36]. These results were generally similar to those found in more extensive and time-intensive multicomponent feedback interventions [37, 38].

As the number of publications focused on computerized PNF effectiveness has increased in recent years, an empirical review of these studies is needed to assess the utility of PNF as a stand-alone computer-based program. Therefore, the purpose of the present study was to summarize available research and to perform a meta-analytic review of computer-delivered stand-alone PNF interventions for college student drinking. Study objectives were to (1) compare effects of gender-neutral and gender-specific PNF on college student drinking, (2) examine the impact of maturation on magnitude of intervention effects, and (3) analyze effects of stand-alone PNF on alcohol-related harms.

## Method

### Sample of Studies

We conducted a three-tier literature search to identify relevant studies. First, we searched electronic databases (PsycINFO, PubMed, MEDLINE, Education Resources Information Center (ERIC), Database of Abstracts of Reviews of Effects (DARE), Cumulative Index to Nursing and Allied Health Literature (CINAHL), Cochrane Central Register of Controlled Trials (CENTRAL), and ProQuest Dissertations and Theses) using a Boolean search strategy with the following search terms: (alcohol OR drink* OR binge) AND (college* OR university) AND (intervention OR prevention OR treatment OR feedback) AND (norm* OR personal* OR individual). Second, we reviewed references of relevant articles and empirical reviews retrieved from database searches. Third, we conducted a forward literature search for publications that cited relevant articles and reviews. The first author (KBD) and a trained bachelor-level research assistant completed the search independently. Finally, we contacted prominent researchers for unpublished findings relevant to this study. Studies meeting selection criteria and available by November 2014 were included.

### Selection Criteria

**Fig 1:**
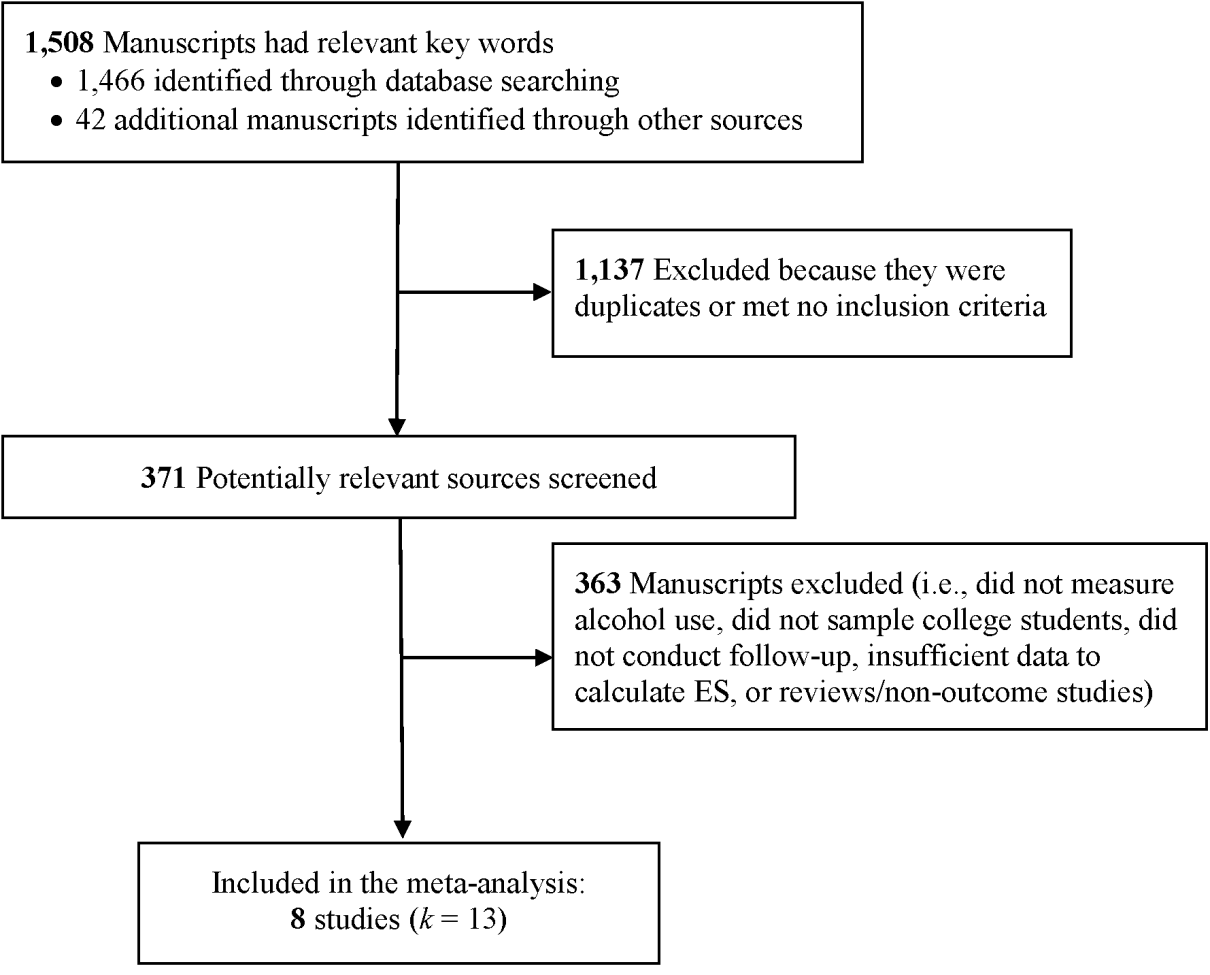
Study flow diagram.

Studies were included if they (a) examined a stand-alone PNF drinking intervention; (b) used a college student sample; (c) included a control condition; (d) reported outcomes for drinking norms and actual drinking behavior; (e) used a pre-post experimental design with a minimum of 28-days between baseline and follow-up; and (f) provided adequate information for effect size (ES) calculation. Studies were excluded if they (a) reported additional intervention components (e.g., alcohol education, protective behavioral strategies, motivational interviewing); (b) used a non-college student sample; or (c) did not report actual drinking outcomes. We requested additional data necessary for inclusion from one primary author but did not receive a response; therefore, the study was excluded from the analysis. All studies were published in English and none were excluded for language. Eight studies (13 interventions) completed between 2004 and 2014 were included, with a total of 2,050 participants (Fig 1) [22, 24–30].

### Coding and Reliability

We developed a comprehensive coding manual to systematically extract study-level and effect-size level data from each study (S1 Appendix). The first author (KBD) and two trained bachelor-level research assistants independently coded sample characteristics (e.g., sex, ethnicity), intervention details (e.g., feedback format, setting, normative referent), and methodology. We coded study design features to assess study quality, including methods for condition assignment, attrition rates, and baseline differences. We assessed risk-of-bias using the assessment tool provided by the Cochrane Collaboration, which includes ratings on each of the following domains: (1) Random sequence generation; (2) Allocation concealment; (3) Blinding of participants and personnel; (4) Blinding of outcome assessment; (5) Incomplete outcome data; (6) Selective reporting; and (7) Other sources of bias [39]. Intercoder reliability was high (Cohen’s *k* = .84). We resolved coding disagreements through discussion.

### Study Outcomes

We calculated effect size estimates for alcohol consumption and alcohol-related problems. When more than one drinking outcome was reported we calculated a separate ES for each outcome. Due to the small sample of studies included in this meta-analysis and variability in drinking outcomes reported across studies, there was insufficient statistical power to warrant separate analyses for each drinking outcome. *Drinks per week* was the only drinking outcome reported consistently across all studies, therefore it was used as the alcohol consumption ES estimate [40]. When multiple follow-ups were reported, the first follow-up was used in analyses.

### Effect Size Derivation

Between-group ESs were calculated as the standardized mean difference in change scores between treatment and control groups divided by the pooled standard deviation (Cohen’s *d*_between_) [41]. We used this formula because it accounts for pretest-posttest correlation (*r*) in independent-groups repeated measures designs [42]. When pre-post correlations were not reported, we used a correlation of 0.6 for within-group comparisons. We calculated this pre-post correlation estimate using an existing dataset of college student drinkers provided by the second author (MED).

Within-group ESs were calculated for each treatment and control group when sufficient data were reported. We calculated within-group ES estimates as the raw mean difference between baseline and follow-up divided by the pooled within-groups SD (*D*_within_). We compared the within-group ES for each intervention condition to its’ respective control condition to examine the impact of maturation on magnitude of intervention effects and to identify potential sources of between-group differences [43].

When means and standard deviations were not reported, we calculated ESs from the available statistics (e.g., *t*-value). Positive ESs indicate a reduction in drinking and alcohol-related problems from baseline to follow-up for participants receiving PNF compared to control group participants. We used inverse variance weighting which allocates ES weights based on standard errors, with more precise ES estimates receiving greater weights [44]. An effect size of 0.2 can be interpreted as small, 0.5 as medium, and 0.8 as large [45]. We conducted separate analyses for gender-specific PNF and gender-neutral PNF when both interventions were compared to the same control group within a study. The assumption of independence precluded use of one analysis to compare multiple interventions to the same control condition [46].

### Statistical analyses

All analyses were conducted using Comprehensive Meta-Analysis ™ [47]. Weighted mean ES estimates (*d*) were calculated using random-effects procedures [47]. The random effects model assumes between-study variance and treats each study as a sample from a population of studies. This model estimates the mean of the distribution of effects, while accounting for differences between studies [47].

Moderator analyses were conducted for variables identified *a priori* [48]. Analysis of variance (ANOVA) was used to analyze categorical variables (e.g., publication type) and meta-regression was used to analyze continuous variables (e.g., length of time until follow up). Power was calculated using random effects method suggested by Hedges and Pigott [49].

## Results

### Descriptives

Descriptive characteristics for the eight studies are provided in Table 1. Publication years ranged from 2004 to 2014. Seven studies were conducted in the United States and one study was conducted in Canada. Four studies were conducted at large public universities, three at mid-size public universities, and one was conducted at two schools (a large public university and a mid-size private university). The analysis included a total of 2,050 participants (PNF *n* = 1,181; control *n* = 869). There were a total of six gender-neutral PNF conditions and seven gender-specific PNF conditions. Five studies used an assessment-only control condition and three studies used an attention-matched control condition.

**Table 1:** Study Descriptives.

Study	Groups (*N*)	Modality	FU Wks	Included sample	*M* age (SD)	Sample	Attrition
Curtis (2005)	GS (34); A/O control (47)	W	6	81	20.5 (1.9)	Undergraduates at a large public university in Canada; 60.0% female	14.6%
**LaBrie, Lewis, Atkins, Neighbors, Zheng, Kenney, Napper, Walter, Kilmer, Hummer, Grossbard, Ghaidarov, Desai, Lee, and Larimer (2013)**	GS (184); GN (187); attention control (184)	W	4	555	19.92 (1.3)	Undergraduates from registrar list at 2 west coast universities in US (large public ~30,000 enrolled; mid-size private ~6,000 enrolled); 75.7% Caucasian; 56.7% female	10.3%*
**Lewis (2005)—Females**	GS (32); GN (39); A/O control (27)	C	4	98	20.01 (1.79)	Undergraduates in psychology course at midsized Midwest university in US; 97.3% Caucasian; 54.6% female	11%*
**Lewis (2005)—Males**	GS (33); GN (21); A/O control (30)	C	4	84	20.01 (1.79)	Undergraduates in psychology course at midsized Midwest university in US; 97.3% Caucasian; 54.6% female	11%*
**Lewis, Neighbors, Oster-Aaland, Kirkeby, Larimer (2007)**	GS (75); GN (82); A/O control (88)	C	20	245	18.53 (2.04)	Freshmen in orientation course at midsized Midwest university in US; 99.6% Caucasian; 52.24% female	14.7%
**Lewis, Patrick, Litt, Atkins, Kim, Blayney, Norris, George, Larimer (2014)**	GS (119); attention control (121)	W	12	240	20.08 (1.48)	Undergraduates contacted via registrar list at large Northwest university in US; 70.0% Caucasian; 57.6% female	9.6%*
**Neighbors, Jensen, Tidwell, Walter, Fossos, Lewis (2011)**	GS (141); attention control (140)	W	12	281	Not reported	Freshmen and sophomores at large Northwest university in US; 52.5% Asian, 32.9% Caucasian; 60.8% female	4.2%*
**Neighbors, Larimer, Lewis (2004)**	GN (126); A/O control (126)	C	12	252	18.5 (1.24)	Psychology students–large Northwest university in US; 79.5% Caucasian; 58.7% female	21%
**Neighbors, Lewis, Bergstrom, Larimer (2006)**	GN (108); A/O control (106)	C	8	214	19.67 (2.02)	Psychology students at midsized Midwest university in US; 98.04% Caucasian; 55.6% female; 59.80% freshmen, 25.00% sophomores, 9.31% juniors, 5.88% seniors	13.6%

*Note*.

* denotes studies from which data from unrelated treatment groups were excluded.

GN = gender-neutral norms; GS = gender-specific norms; A/O = assessment-only control group; W = web-based in non-structured setting; C = computer-based in structured setting with paper printout; FU Wks = number of weeks from baseline to first follow-up.

Risk of bias varied among studies (see S1 Table), with 5 studies reporting adequate sequence generation, 2 reporting allocation concealment, 7 reporting low risk for selective reporting, and 8 studies with unclear reporting for blinding of outcome assessment. Attrition rates ranged from 4.2% to 21%.

### Intervention Effects on Drinking

**Fig 2:**
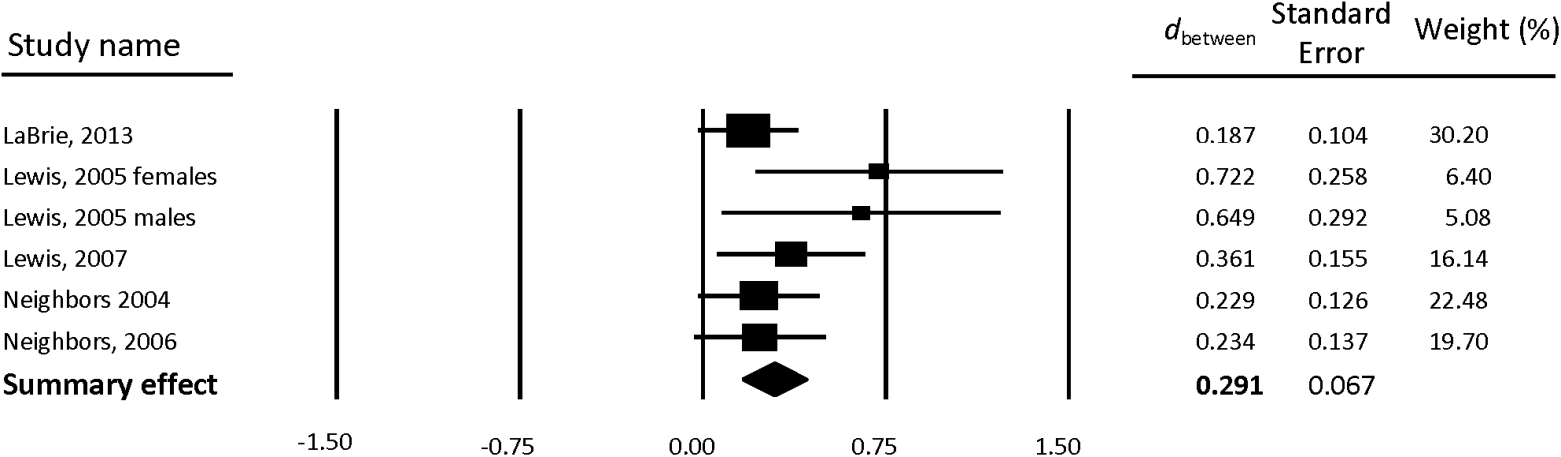
Forest plot of gender-neutral PNF between-group effects (*d*_between_) for drinks per week.

**Fig 3:**
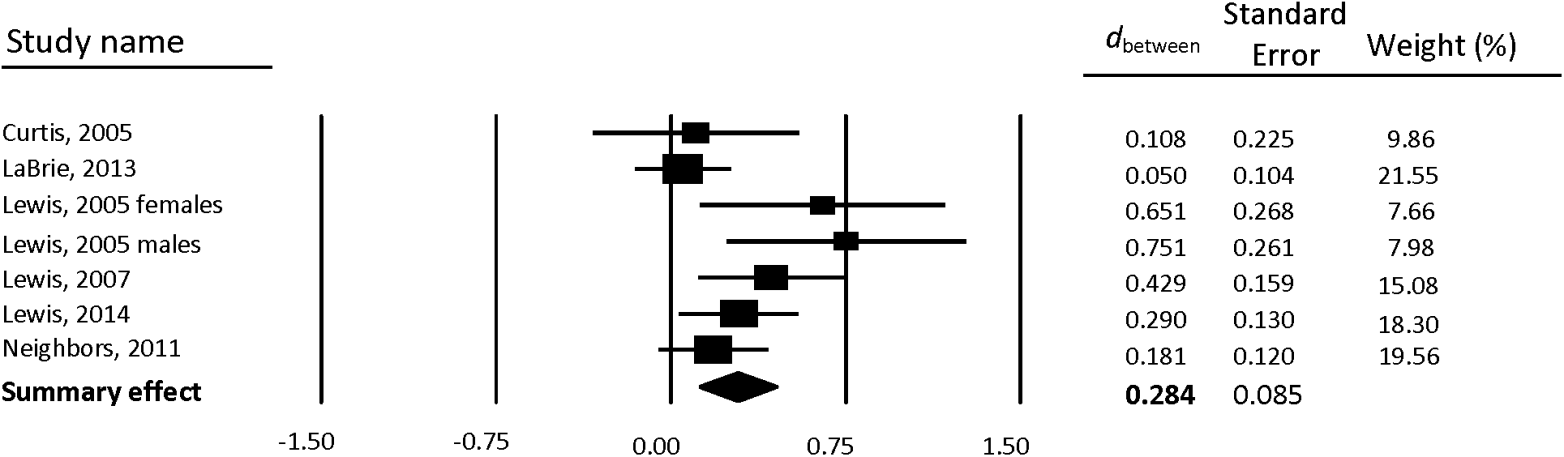
Forest plot of gender-specific PNF between-group effects (*d*_between_) for drinks per week.

Between-group weighted mean ESs are reported separately for gender-neutral PNF (Table 2) and gender-specific PNF (Table 3). Forest plots also are provided separately for gender-neutral PNF (Fig 2) and gender-specific PNF (Fig 3). Compared to control participants, students who received PNF reported a greater reduction in drinking from baseline to follow-up. Results were similar for both gender-neutral PNF (*d* = 0.291, 95% CI [0.159, 0.423]) and gender-specific PNF (*d* = 0.284, 95% CI [0.117, 0.451]).

**Table 2:** Gender-neutral PNF between-group weighted mean ESs for drinks per week.

			Sample size		
Study	Subgroup	FU Wks	PNF	Control	*d*_between_ (95% CI)
**LaBrie, Lewis, Atkins, Neighbors, Zheng, Kenney, Napper, Walter, Kilmer, Hummer, Grossbard, Ghaidarov, Desai, Lee, and Larimer (2013)**		4	187	184	0.187 (-0.017, 0.390)
**Lewis (2005)**	Females	4	39	27	**0.722 (0.216, 1.228)**
**Lewis (2005)**	Males	4	21	30	**0.649 (0.077, 1.220)**
**Lewis, Neighbors, Oster-Aaland, Kirkeby, Larimer (2007)**		20	82	88	**0.361 (0.058, 0.664)**
**Neighbors, Larimer, Lewis (2004)**		12	126	126	0.229 (-0.018, 0.477)
**Neighbors, Lewis, Bergstrom, Larimer (2006)**		8	108	106	0.234 (-0.035, 0.502)
**Summary effect (*k* = 6)**			563	561	**0.291 (0.159, 0.423)**

*Note*. Positive between-group effect sizes (*d*_between_) indicate improved outcome for treatment groups compared to control. Bold font indicates statistically significant weighted mean ES. PNF = Personalized Normative Feedback; FU Wks = number of weeks from baseline to first follow-up; *k* = number of interventions; CI = confidence interval.

**Table 3:** Gender-specific PNF between-group weighted mean ESs for drinks per week.

			Sample size		
Study	Subgroup	FU Wks	PNF	Control	*d*_between_ (95% CI)
**Curtis (2005)**		6	34	47	0.108 (-0.334, 0.549)
**LaBrie, Lewis, Atkins, Neighbors, Zheng, Kenney, Napper, Walter, Kilmer, Hummer, Grossbard, Ghaidarov, Desai, Lee, and Larimer (2013)**		4	184	184	0.050 (-0.154, 0.255)
**Lewis (2005)**	Females	4	32	27	**0.651 (0.125, 1.176)**
**Lewis (2005)**	Males	4	33	30	**0.751 (0.239, 1.262)**
**Lewis, Neighbors, Oster-Aaland, Kirkeby, Larimer (2007)**		20	75	88	**0.429 (0.117, 0.741)**
**Lewis, Patrick, Litt, Atkins, Kim, Blayney, Norris, George, Larimer (2014)**		12	119	121	**0.290 (0.035, 0.544)**
**Neighbors, Jensen, Tidwell, Walter, Fossos, Lewis (2011)**		12	141	140	0.181 (-0.053, 0.416)
**Summary effect (k = 7)**			618	637	**0.284 (0.117, 0.451)**

*Note*. Positive between-group effect sizes (*d*_between_) indicate improved outcome for treatment groups compared to control. Bold font indicates statistically significant weighted mean ES. PNF = Personalized Normative Feedback; FU Wks = number of weeks from baseline to first follow-up; *k* = number of interventions; CI = confidence interval.

Within-group raw mean differences (*D*_within_) are provided separately for gender-neutral PNF (Table 4) and gender-specific PNF (Table 5). Overall, compared to baseline, students who received gender-neutral PNF (*k* = 5) reported 3.027 (95% CI [2.171, 3.882], *p* < .001) fewer drinks per week at first follow-up. Results were similar for gender-specific PNF (*k* = 5), with students reporting 3.089 (95% CI [0.992, 5.186], *p* = .004) fewer drinks per week at first follow-up, compared to baseline.

**Table 4:** Gender-neutral PNF within-group effects for drinks per week.

			Sample size		GN PNF	Control
Study	Subgroup	FU Wks	PNF	Control	*D*_within_	*D*_within_
**LaBrie, Lewis, Atkins, Neighbors, Zheng, Kenney, Napper, Walter, Kilmer, Hummer, Grossbard, Ghaidarov, Desai, Lee, and Larimer (2013)**		4	187	184	**1.900 (0.746, 3.054)**	0.300 (-0.962, 1.562)
**Lewis (2005)**	Females	4	39	27	**3.650 (1.807, 5.493)**	-0.180 (-2.447, 2.087)
**Lewis (2005)**	Males	4	21	30	**4.440 (0.516, 8.364)**	-1.450 (-4.825, 1.925)
**Neighbors, Larimer, Lewis (2004)**		12	126	126	**3.410 (2.061, 4.759)**	**1.460 (0.003, 2.917)**
**Neighbors, Lewis, Bergstrom, Larimer (2006)**		8	108	106	**3.600 (1.784, 5.416)**	1.280 (-0.638, 3.198)
**Summary effect (k = 5)**			563	561	**3.027 (2.171, 3.882)**	0.642 (-0.135, 1.420)

*Note*. Positive *D*_within_ indicates a reduction in drinks per week from baseline to follow-up. Bold font indicates statistically significant weighted mean difference. *D*_within_ = raw mean difference; GN = gender-neutral; PNF = Personalized Normative Feedback; *k* = number of interventions; FU Wks = number of weeks from baseline to first follow-up; CI = confidence interval.

**Table 5:** Gender-specific PNF within-group effects for drinks per week.

			Sample size		GS PNF	Control
Study	Subgroup	FU Wks	PNF	Control	*D*_within_	*D*_within_
**Curtis (2005)**		6	34	47	1.000 (-1.202, 3.202)	0.100 (-2.087, 2.287)
**LaBrie, Lewis, Atkins, Neighbors, Zheng, Kenney, Napper, Walter, Kilmer, Hummer, Grossbard, Ghaidarov, Desai, Lee, and Larimer (2013)**		4	184	184	0.800 (-0.403, 2.003)	0.300 (-0.962, 1.562)
**Lewis (2005)**	Females	4	32	27	**3.830 (1.412, 6.248)**	-0.180 (-2.447, 2.087)
**Lewis (2005)**	Males	4	33	30	**5.390 (2.400, 8.380)**	-1.450 (-4.825, 1.925)
**Lewis, Patrick, Litt, Atkins, Kim, Blayney, Norris, George, Larimer (2014)**		12	119	121	**5.010 (3.393, 6.627)**	**2.470 (0.929, 4.011)**
**Summary effect (k = 5)**					**3.089 (0.992, 5.186)**	0.557 (-0.663, 1.778)

*Note*. Positive *D*_within_ indicates a reduction in drinks per week from baseline to follow-up. Bold font indicates statistically significant weighted mean difference. *D*_within_ = raw mean difference; GS = gender-specific; PNF = Personalized Normative Feedback; *k* = number of interventions; FU Wks = number of weeks from baseline to first follow-up; CI = confidence interval.

### Intervention Effects on Alcohol-related Harms

Between-group weighted mean ESs for alcohol-related harms are reported in Table 6. Compared to control participants, students who received PNF reported a greater reduction in alcohol-related harms from baseline to follow-up, though observed effects were minimal (*d* = 0.157, 95% CI [0.037, 0.278], *p* = .010).

**Table 6:** Between-group weighted mean effects for alcohol-related harms.

Study	Harms Measure	*d*_between_ (95% CI)
Curtis (2005)	SIP	0.196 (-0.246, 0.639)
LaBrie, Lewis, Atkins, Neighbors, Zheng, Kenney, Napper, Walter, Kilmer, Hummer, Grossbard, Ghaidarov, Desai, Lee, and Larimer (2013)	RAPI	0.175 (-0.060, 0.409)
Lewis, Patrick, Litt, Atkins, Kim, Blayney, Norris, George, Larimer (2014)	BYAACQ	0.134 (-0.119, 0.387)
Neighbors, Larimer, Lewis (2004)	RAPI	0.127 (-0.120, 0.375)
Neighbors, Lewis, Bergstrom, Larimer (2006)	RAPI	0.183 (-0.088, 0.454)
Summary effect (*k* = 5)		**0.157 (0.037, 0.278)**

*Note*. Positive between-group effect sizes (*d*_+_) indicate improved outcome for treatment groups compared to control. Bold font indicates statistically significant weighted mean ES. *k* = number of interventions; CI = confidence interval; SIP = Short Index of Problems; RAPI = Rutgers Alcohol Problems Index; BYAACQ = Brief Young Adult Alcohol Consequences Questionnaire.

### Moderator Analyses

The *Q* statistic was calculated to measure the presence or absence of homogeneity. A significant *Q*-value indicates that homogeneity is not present and suggests that there may be heterogeneity. The *I* ^2^ statistic provides an estimate of heterogeneity ranging from 0 to 100%. Larger values of *I* ^2^ indicate a greater degree of heterogeneity, with 25% interpreted as low, 50% as moderate, and 75% as high [50]. For gender-neutral PNF (*k* = 6) the Q-value was non-significant (*Q* = 4.695, *p* = .454) and *I* ^2^ = 0.000, suggesting homogeneity. For gender-specific PNF (*k* = 7) the Q-value was non-significant (*Q* = 10.863, *p* = .093) and *I* ^2^ = 44.768, suggesting a low to moderate degree of heterogeneity. Studies were examined and one outlier was identified. Sensitivity analyses revealed that the outlier had little impact on the summary effect. Excluding the outlier would not change the interpretation of the findings, therefore, in order to conserve power we did not exclude it from main effects analyses. We conducted moderator analyses for variables identified a priori. Moderator analyses for both gender-neutral and gender-specific PNF were non-significant for all moderator variables examined (i.e., modality, normative referent, control type, follow-up time, publication type, and sample).

### Publication Bias

We conducted fail-safe analyses to estimate the number of missing studies with null findings that would nullify the observed summary effect [51]. Results indicated that 36 studies would be necessary to nullify the findings for both gender-neutral and gender-specific PNF. We conducted trim-and-fill analyses to assess and adjust for publication bias [52]. Results suggested two missing studies. Weighted mean ESs adjusted for publication bias were similar to unadjusted ESs for both gender-neutral PNF (observed *d* = 0.291, 95% CI [0.159, 0.423]; adjusted *d* = 0.243, 95% CI [0.083, 0.403]) and gender-specific PNF (observed *d* = 0.284, 95% CI [0.117, 0.451]; adjusted *d* = 0.206, 95% CI [0.024, 0.387]). These results indicate that including two missing studies would not change overall findings or implications of this meta-analysis.

## Discussion

Results from this meta-analysis indicate that computer-delivered PNF is an effective stand-alone approach for reducing college student drinking, but has minimal impact on alcohol-related harms. Overall, effect sizes were small but significant for alcohol use and less than small for alcohol-related harms. Results were consistent regardless of intervention setting. These findings suggest that computer-delivered PNF is equally effective when completed in a structured setting or when completed in a non-structured setting. Foxcroft and colleagues reported similar findings in their recent systematic review of social norms approaches for college student drinking [18]; however, they concluded that effects were not clinically significant. Though PNF may be limited in clinical significance as a primary intervention, the observed effects on drinking are clinically relevant when PNF is examined from a public health perspective as a preventive approach.

Outcome research typically examines differences between treatment and control groups following a treatment, with between-group differences representing treatment effects. While this methodological approach is appropriate for treatment outcome studies, it is not ideal for prevention research. The “prevention paradox” asserts that the aim of prevention is to improve global outcomes by causing small changes among a large portion of a given population. As such, prevention programs would be expected to improve population-level outcomes, with relatively small improvements on an individual level [53]. Given the substantial interpretive differences for treatment versus prevention effects, it is imperative that researchers consider carefully the aims and anticipated outcomes of intervention studies in order to measure and report outcomes accurately. For example, in a college-based prevention study measuring drinking outcomes as drinks per week, we would not predict a large effect for drink reductions. Instead, we would anticipate a slower increase in drinking at follow-up for students receiving prevention compared to students in a control condition. Further, although *drinks per week* provides a common metric of overall drinking quantity, it is not the best measure of drinking patterns among college students, which typically consist of periodic binge drinking episodes rather than steady frequent drinking. Although *number of binge drinking episodes* is commonly reported, this measure provides categorical data with limited utility in allowing researchers to understand an intervention’s impact on drinking. Rather than reporting categorical data, it would be more useful to report the actual number of drinks consumed during each binge drinking episode or other continuous measures of drinking behavior. Appropriate outcome reporting has important implications for interpreting effects in intervention outcome studies. For example, a reduction of four drinks per week will be interpreted much differently if the reduction occurs during a single binge drinking episode compared to a reduction of four drinks that occurs across several drinking occasions. It is imperative that researchers assess outcomes appropriately for the population of interest, rather than selecting a measure solely because it is a common metric.

There is a natural maturation process that seems to occur for most college student drinkers. Research has shown that first-year college students are a particularly high-risk group and that there is a natural “maturing out” process, whereby high-risk drinking tends to decrease over time [54]. Results of the present study suggest that PNF may expedite this process. When comparing PNF within-group effects to within-group effects of control participants, drinking tends to decline between baseline and first follow-up with a more pronounced effect for students who received PNF. Though the overall within-group effect for controls is not statistically significant, the pattern of maturation is evident (see Tables 4 & 5). More robust findings may have been observed if longer-term follow-up data had been included. However, only five studies in the present meta-analysis included multiple follow-up time points therefore there was not adequate power to conduct separate analyses at each time point. Longitudinal research is needed to examine the degree to which PNF attenuates drinking among college students and to assess the degree to which PNF may accelerate the “maturing out” process. This maturation effect will not be apparent when outcomes are analyzed using only between-groups, post-test mean differences. To study the impact of PNF on drinking maturation, researchers should incorporate statistical approaches that examine changes between and within groups over time.

In addition to examining the potential positive effects of PNF, future research should consider the limitations and problems with PNF. Given the proliferation of web-based intervention approaches, researchers must consider whether students are actually viewing and processing the intervention content. According to a recent study by Lewis and Neighbors, students reported engaging in other activities while completing web-based interventions [55]. This finding highlights the need for researchers to develop interventions that garner engagement, particularly if they are to be delivered in non-structured settings. This is important because web-based interventions provide an efficient and cost-effective approach to delivering interventions to college students.

Environmental context is an additional issue to consider with web-based interventions. Findings from a recent study by Monk and Heim [56] indicated that environmental context may moderate alcohol-related cognitions, including alcohol norms. Students in this study reported higher positive expectancies and lower drink refusal self-efficacy when questionnaires were administered in a bar setting compared to a lecture hall [56]. As web-delivered interventions become more widespread, it is essential that researchers consider the impact of environmental context on both intervention development and outcome assessment. This is particularly important when researching alcohol norms and expectancies, as alcohol-related cognitions are the target for these intervention approaches.

Researchers have also voiced concerns about a potential “boomerang effect” for low risk drinkers that may lead them to drink more after receiving feedback stating that they consume less alcohol than peers. In a recent study, Prince and colleagues assessed for a boomerang effect among low-risk drinkers by examining drinking outcomes in feedback-based intervention trials [57]. Computer-delivered stand-alone PNF interventions were used in two of the trials, which included a total of 466 undergraduate students (Trial 1 *n* = 252; Trial 1 *n* = 214). Researchers found no evidence to support a boomerang effect for low risk drinkers [57]. Although this study provides preliminary support for PNF as a universal prevention program, as research on stand-alone PNF expands, continued efforts should be made to assess potential negative outcomes among non-drinkers.

Finally, a major problem remaining in the literature is the overly general and inconsistent terminology used to refer to various interventions with heterogeneous content. Common terms used to describe these interventions include personalized feedback, personalized normative feedback, normative feedback, individualized feedback, and multicomponent feedback, among others. Inconsistent use of these terms may lead to inaccurate interpretations of intervention effects when the same term is used to identify various combinations of intervention components. Future research in this area should consider adopting a consistent set of terms to identify specific interventions and components.

### Limitations

There are several notable limitations of this study. First, only eight primary studies were included in this meta-analysis. With such a small sample of studies, generalizability of these findings may be limited and results must be interpreted accordingly. Second, all eight studies were conducted in North America. Given this geographic limitation in sampling, the findings of this meta-analysis may not be generalizable and results may differ in other countries. Third, given the relatively recent advent of stand-alone PNF, much of the research has been conducted by a small pool of researchers which could further limit generalizability. Fourth, by design, meta-analyses are limited by the studies and data made available for inclusion. Substantial efforts were made to conduct a thorough, exhaustive literature search and to seek additional unpublished data through direct contact attempts with prominent researchers. Fifth, outcomes and moderators were limited to the data included in the primary studies; therefore, it was not possible to examine the impact of other variables of interest (e.g., social reasons for drinking, long-term follow-up).

Overall, findings of this meta-analysis suggest that stand-alone PNF is promising as a universal prevention approach for college student drinking. Given the limited pool of primary studies, it is recommended that this review be updated when additional studies are available for inclusion. Despite the limitations, this study offers an empirical summary of computer-delivered stand-alone PNF for college student drinking and provides a foundation for future prevention research.

## Supporting Information

S1 PRISMA Checklist PRISMA Checklist. (PDF)

S1 Appendix Coding Manual. (PDF)

S1 Table Risk of Bias. (PDF)
