# Supplementary material for: Stand-Alone Personalized Normative Feedback for College Student Drinkers: A Meta-Analytic Review, 2004 to 2014
Source: PLoS One. 2015 Oct 8;10(10):e0139518. doi: 10.1371/journal.pone.0139518 (PMC4598082; doi:10.1371/journal.pone.0139518)
Supplement: S1 Appendix — (PDF) [file pone.0139518.s002.pdf]

__ __ Coder ID

STUDY-LEVEL CODING FORM

Reference: ____

__ __ __ __ 1. Study ID number [STUDYID]

__ 2. Type of publication [PUBTYPE]

1 journal article

2 dissertation

3 other (specify): _______________________

__ __ __ __ 3. Publication year [PUBYEAR]

Sample Descriptors

__ __ . __ __ 4. Mean age [MEANAGE]

__ 5. Predominant race/ethnicity [RACE]

1 >60% White

2 >60% Black

3 >60% Hispanic

4 >60% Other

5 mixed, none more than 60%

6 mixed, cannot estimate proportion

9 insufficient information

__ 6. Predominant sex [SEX]

1 <5% female

2 5%-25% female

3 26%-49% female

4 50% female

5 51%-74% female

6 75%-95% female

7 >95% female

9 insufficient information

__ 7. Subject sample [SUBJECTS]

1 General college student sample

2 First-year (freshman) students

3 Freshman and sophomore students, combined

4 other (specify): _______________

__ 8. Participant recruitment method [RECRUIT]

1 Email from registrar list

2 Psychology classes

3 Other ______________

Research Design Descriptors

__ 9. Type of assignment to conditions [ASSIGN]

1 random after matching, stratification, blocking, etc.

2 random, simple

3 nonrandom

4 other (specify): _____________

5 insufficient information

__ 10. Overall confidence of judgment on how subjects were assigned [CRASSIGN]

1 very low (little basis)

2 low (guess)

3 moderate (weak inference)

4 high (strong inference)

5 very high (explicitly stated)

__ 11. Baseline differences between treatment and control groups [PREDIFFS]

1 negligible differences, judged unimportant

2 some differences, judged of uncertain importance

3 some differences, judged important

__ __ __ __ 12. Treatment group sample size at start of study (completed baseline) [ORIG_TXN]

__ __ __ __ 13. Control group sample size at start of study (completed baseline) [ORIG_CN]

__ __ __ __ 14. Total sample size at start of study (completed baseline) [TOTALN]

Intervention Descriptors

__ 15. Type of administration [ADMIN]

1 self-guided

2 provider-guided

__ 16. Intervention modality [MODALITY]

1 computer-based in a structured setting (lab/clinic), with paper feedback

2 computer-based in a structured setting (lab/clinic), without paper feedback

3 web-based, non-structured setting

4 paper-based, in a structured setting (lab/clinic)

5 verbal, in a structured setting (lab/clinic)

6 other (specify): ___________________________

__ 17. Normative referent group [NORMREF]

1 Gender-neutral

2 Gender-specific

3 Other ___________________

4 Unknown

__ 18. Type of control group [CGTYPE]

1 waitlist, assessment only

2 attention-matched (not related to alcohol use)

3 treatment as usual, minimal content (alcohol education)

4 treatment as usual, brief therapy with provider

5 other (specify) ____________________________

__ __ Coder ID

EFFECT SIZE LEVEL CODING FORM

__ __ __ __ 1. Study ID number [STUDYID]

__ __ 2. Effect size type [ESTYPE]

1 drinks per week

2 drinks per sitting

3 peak drinks

4 frequency

6 drinking composite, quantity-frequency measure

7 harms

8 norms

Dependent Measures Descriptors

__ 3. Follow-up type [FU_TYPE]

1 posttest comparison (first follow-up post-intervention)

2 follow-up comparison (any additional follow-ups)

3 final follow-up comparison (use this if only one follow-up)

__ __ __ 4. Time since baseline, in weeks [FU_WKS]

__ 5. Category of outcome construct [OUTCOME]

1 drinking outcome, single measure (e.g., drinks per week, peak BAC, frequency)

2 drinking outcome, combined (e.g., ACI, quantity-frequency measure, composite)

3 harms measure (BYAACQ, RAPI, SIP)

4 norms measure

Effect Size Data

__ 6. Type of data effect size based on [ESDATA]

1 adjusted means and standard deviations

2 raw means and standard deviations

3 adjusted means and standard errors

4 means and standard errors

5 *t*-value or *F*-value

6 ratio measure

7 mean difference

8 other ________________________

__ __ __ __ 7. Page number where effect size data found [PAGENUM]

Sample Size

__ __ __ 8a. Treatment group sample size at baseline [TXN]

__ __ __ 8b. Control group sample size baseline [CGN]

Answer the following questions to help select the appropriate ES calculation for each outcome:

1) Are the means and standard deviations/standard errors provided for both the treatment and control group?

If NO: Proceed to next question.

If YES: Are M and SD/SE provided for both baseline *and* follow-up?

If YES: Complete both sections of A1 (for SD) or A2 (for SE) below.

If NO: Complete "Follow-up (Post-test)" section of A1 (for SD) or A2 (for SE) below.

2) Is the *t*-value of the outcome provided comparing the treatment and control group?

IF NO: Proceed to next question.

IF YES: Enter the t-value under section B below, then use the calculator found at:

http://www.campbellcollaboration.org/escalc/html/EffectSizeCalculator-SMD2.php

to calculate Cohen's *d*. Enter *d* and variance under "Calculated effect size".

3) Is the mean difference (*d*) provided comparing the treatment and control group?

IF NO: Enter available data in appropriate section below.

IF YES: Complete section D below AND enter *d* value under "calculated effect size"

A1 ***Means and Standard Deviations***

Baseline (Pre-test)

__ __ __ __ 9a. Treatment group mean at baseline [TXMEANPRE]

__ __ __ __ 9b. Control group mean at baseline [CGMEANPRE]

__ __ __ __ 9c. Treatment group standard deviation at baseline [TXSDPRE]

__ __ __ __ 9d. Control group standard deviation at baseline [CGSDPRE]

Follow-up (Post-test)

__ __ __ __ 9e. Treatment group mean at follow-up [TXMEANPOST]

__ __ __ __ 9f. Control group mean at follow-up [CGMEANPOST]

__ __ __ __ 9g. Treatment group standard deviation at follow-up [TXSDPOST]

__ __ __ __ 9h. Control group standard deviation at follow-up [CGSDPOST]

__ __ __ __ 9i. Pre-post correlation (if not reported enter 0.6) [PPCORR]

A2 ***Means and Standard Errors*** ****Calculate SDs using Excel spreadsheet*

Baseline (Pre-test)

__ __ __ __ 10a. Treatment group mean at baseline [TXMEANPRE]

__ __ __ __ 10b. Control group mean at baseline [CGMEANPRE]

__ __ __ __ 10c. Treatment group standard error at baseline [TXSEPRE]

Calculated SD ____________________ [TXPRE_CALCSD]

__ __ __ __ 10d. Control group standard error at baseline [CGSEPRE]

Calculated SD ____________________ [CGPRE_CALCSD]

Follow-up (Post-test)

__ __ __ __ 10e. Treatment group mean at follow-up [TXMEANPOST]

__ __ __ __ 10f. Control group mean at follow-up [CGMEANPOST]

__ __ __ __ 10g. Treatment group standard error at follow-up [TXSEPOST]

Calculated SD ____________________ [TXPOST_CALCSD]

__ __ __ __ 10h. Control group standard error at follow-up [CGSEPOST]

Calculated SD ____________________ [CGPOST_CALCSD]

__ __ __ __ 10i. Pre-post correlation (if not reported enter 0.6) [PPCORR]

B ***Significance Tests***

__ __ __ __ 11a. *t*-value [T_VALUE]

__ __ __ __ 11b. *F*-value (*df* for the numerator must = 1) [F_VALUE]

__ __ __ __ 11c. Chi-square value (*df* = 1) [CHISQUARE]

C ***Ratios***

__ __ __ __ 12a. Rate ratio [RR_VALUE]

__ __ __ __ 12b. Rate ratio confidence interval [RR_CI]

D ***Mean difference (d)***

__ __ __ __ 13a. Mean difference (*d*)

__ __ __ 13b. Treatment group sample size at baseline [TXN]

__ __ __ 13c. Control group sample size at baseline [CGN]

Calculated Effect Size

__ __ __ __ 14. Effect size (Cohen's *d*) [ES]

__ __ __ __ 15. Effect size variance [ESVAR] – if calculated

__ 16. Confidence rating in effect size computation [CR_ES]

1 highly estimated

2 moderate estimation

3 some estimation

4 slight estimation

5 no estimation
