# Supplementary material for: Stand-Alone Personalized Normative Feedback for College Student Drinkers: A Meta-Analytic Review, 2004 to 2014
Source: PLoS One. 2015 Oct 8;10(10):e0139518. doi: 10.1371/journal.pone.0139518 (PMC4598082; doi:10.1371/journal.pone.0139518)
Supplement: S1 Table — (PDF) [file pone.0139518.s003.pdf]

	Random sequence generation (selection bias)	Allocation concealment (selection bias)	Blinding of participants and personel (performance bias)	Blinding of outcome assessment (detection bias)	Incomplete outcome data (attrition bias)	Selective reporting (reporting bias)
Curtis 2005	+	+	-	?	+	+
LaBrie 2013	+	?	-	?	+	+
Lewis 2005	+	?	-	?	+	+
Lewis 2007	?	?	-	?	+	+
Lewis 2014	+	+	-	?	+	+
Neighbors 2011	+	?	-	?	+	-
Neighbors 2004	?	?	-	?	+	+
Neighbors 2006	?	?	-	?	+	+

+ = high quality (low risk of bias); - = low quality (high risk of bias); ? = unclear
